# N-Type Coating of Single-Walled Carbon Nanotubes by Polydopamine-Mediated Nickel Metallization

**DOI:** 10.3390/nano13202813

**Published:** 2023-10-23

**Authors:** Cordelia Zimmerer, Frank Simon, Sascha Putzke, Astrid Drechsler, Andreas Janke, Beate Krause

**Affiliations:** 1 Institute of Polymer Materials, Leibniz-Institut für Polymerforschung Dresden e.V. (IPF), Hohe Str. 6, 01069 Dresden, Germany; zimmerer@ipfdd.de (C.Z.); putzke@ipfdd.de (S.P.);; 2 Institute of Physical Chemistry and Polymer Physics, Leibniz-Institut für Polymerforschung Dresden e.V. (IPF), Hohe Str. 6, 01069 Dresden, Germany; frsimon@ipfdd.de (F.S.); drechsler@ipfdd.de (A.D.); 3 Institute of Macromolecular Chemistry, Leibniz-Institut für Polymerforschung Dresden e.V. (IPF), Hohe Str. 6, 01069 Dresden, Germany

**Keywords:** thermoelectric, carbon nanotubes, polydopamine, nickel

## Abstract

Single-walled carbon nanotubes (SWCNTs) have unique thermal and electrical properties. Coating them with a thin metal layer can provide promising materials for many applications. This study presents a bio-inspired, environmentally friendly technique for CNT metallization using polydopamine (PDA) as an adhesion promoter, followed by electroless plating with nickel. To improve the dispersion in the aqueous reaction baths, part of the SWCNTs was oxidized prior to PDA coating. The SWCNTs were studied before and after PDA deposition and metallization by scanning and transmission electron microscopy, scanning force microscopy, and X-ray photoelectron spectroscopy. These methods verified the successful coating and revealed that the distribution of PDA and nickel was significantly improved by the prior oxidation step. Thermoelectric characterization showed that the PDA layer acted as a p-dopant, increasing the Seebeck coefficient S of the SWCNTs. The subsequent metallization decreased S, but no negative S-values were reached. Both coatings affected the volume conductivity and the power factor, too. Thus, electroless metallization of oxidized and PDA-coated SWCNTs is a suitable method to create a homogeneous metal layer and to adjust their conduction type, but more work is necessary to optimize the thermoelectric properties.

## 1. Introduction

Applying carbon nanotubes (CNTs) as fillers in metal or other material composites is promising to create materials with unique performance. Metallized CNTs can be considered steady organic metals without the need for further activation by doping or charge transfer to achieve powerful electron carrier mobility [1]. Especially for lightweight metal-matrix composites [2], electrical and thermal conductive adhesives [3], impact protection and vibration damping [4], metallized carbon allotropes have great potential as compatibilized fillers [5,6,7], for sensors [8,9,10], catalysis [8,11], and energy storage [12].

CNTs have exceptional thermal and electrical properties. They are able to generate thermoelectric voltage when a temperature gradient is applied [13] and can thus be used for thermoelectric (TE) applications [14,15,16,17,18,19]. With regard to the thermoelectric performance of CNTs, however, it has to be noted that it is lower than that of traditional TE materials, such as half-Heusler compounds, clathrates, silicides, antimonides, and tellurides [13,20].

The thermoelectric (TE) properties are characterized by the Seebeck coefficient S. This parameter S is calculated from the generated thermoelectric voltage (U) divided by the applied temperature difference (ΔT) (Equation (1)). p-conductive electrical behaviour (conduction based on the directional movement of defect electrons (holes)) is indicated by a positive S-value and n-conductive behaviour (conduction through freely movable electrons) by a negative S-value. The second parameter used is the power factor PF, which is calculated from the product of the squared Seebeck coefficient S and the volume conductivity σ (Equation (2)) [21,22].

(1) $$S=\frac{U}{∆T}$$

(2) $$PF=S^{2}\cdot \sigma$$

Nonoguchi et al. [15] have conducted a screening experiment with a wide range of additives, which include phosphine-containing and imine-containing molecules as dopants for single-walled CNTs (SWCNTs). Different polymeric dopants were described by Piao et al. [14] for the modification of pieces of SWCNT buckypaper by immersing them overnight in the respective solutions. In both papers, it was reported that the additives can change the initial positive S-value of SWCNTs to other positive S-values but also to negative values. Hata et al. [18] showed that the Seebeck coefficient could be reduced from 62.3 µV/K for pure CNTs to values between −30.1 and −44.1 µV/K when nanotubes were wrapped with surfactants. Tzounis et al. [16] described polyetherimide (PEI)-based composites with SWCNTs, whereby the Seebeck coefficient of the pure SWCNTs at 31 µV/K could be increased up to 55 µV/K for a composite containing 4.4 vol% SWCNTs. Mytafides et al. [17] prepared n-type SWCNT films by solution mixing of SWCNTs with cetyltrimethylammonium bromide (CTAB). All these doping methods have in common that the p-conductive properties of the SWCNTs are enhanced or changed to n-conductive by bringing polymers to their surface in the solution.

The preparation of CNT composites faces, however, serious challenges:

- Creating well-dispersed systems in the matrix material;
- ensuring high interfacial adhesion to the matrix material;
- avoiding damage, such as structure defects and shortening, to the CNTs and thus altering their properties during composite preparation, especially for metal matrices.

To fulfil the first two requirements, highly oxidative, toxic, and, in general, environmentally harmful chemicals [23,24,25] or expensive, energy-consuming processes have been widely applied [23,24,25], and result in chemical modifications of the surface to improve the wetting of the CNTs and, therefore, the compatibility with the partner material(s) of the composite. A further disadvantage of chemical surface functionalization is the difficult control of the balance between functionalization and the damage degree of CNTs.

New paths open up with bioinspired concepts in material science. A versatile adhesion promotion concept from the field of synthetic biology has been successfully transferred to the technical sphere in recent years, adapting the mussel adhesive derivative dopamine (DA) as an environmentally friendly bridge between different types of materials [26,27,28]. DA can be applied in a “green” process as a water-based coating. On solid surfaces, it auto-oxidizes spontaneously under oxygen exposure and forms a thin film of oligomers and polymers, the so-called polydopamine (PDA), with high intra- and intermolecular interaction potential [29,30]. The universal adhesion mechanism is explained by the various functional groups of DA and the fact that loosely adhering layers are replaced by DA during its polymerization directly on the surface. This reaction is accompanied by a volume contraction, which leads to interlocking with the surface [28]. In particular, the size of the CNTs and their rod shape geometrically favour stable functionalization with PDA as a closed wrapping. The polymerisation of PDA leads to a covalently cross-linked, enveloping film around the tubular structure, and the shrinkage during polymerisation leads to close contact with the CNT carbon backbone. Furthermore, the high polarity of the hydroxyl groups on the surfaces of PDA leads to a significant change in the electronic properties of the CNTs due to their interaction with π-electrons in the benzene rings of the CNTs [31,32,33]. Studies show that CNTs are capable of absorbing various metal ions in solutions [34,35]. Molecular dynamics simulations have shown that hydroxyl and carboxyl functional groups on the CNT surface lead to more effective adsorption of Cu (II) ions [36].

In general, the contact resistance of the CNTs is affected by any functionalization. Yet, PDA films are only a few molecular layers thick and exhibit redox-active behaviour that enables electron transfer. With PDA coating, high compatibility of the CNTs with and adhesion to metals are expected. PDA functionalization of CNTs was applied for decoration with gold nanoparticles [37] and in electrodeposition composite coating formation [38,39]. Elsewhere, electroless metallization with silver and copper based on PDA as an adhesion promoter was demonstrated on tungsten carbide microparticles and on alumina nanoparticles [40].

Different metallization methods [1,23,24,41,42] such as powder metallurgy techniques, electroless plating, electroplating, physical and chemical vapour deposition, flame-, arc-, and supersonic spraying, make a colourful bouquet for different metals or applications [5]. The most well-known method is autocatalytic electroless plating. It has high mass production importance because it is scalable and straightforward in a non-vacuum environment. Metals such as those given in Table 1 can be processed from aqueous solutions under suitable conditions (reducing agent, temperature, bath composition, pH, and corresponding catalytic surface) [11]. Especially Ni possesses technical relevance because the plated layers are almost not porous, smooth and hard, and show more uniform thickness and better corrosion resistance. Against the background of the suitability of the metals as thermoelectric materials, the Seebeck coefficients S of the pure metals are given in Table 1. Low, mostly single-digit values with positive and negative signs are observed for the metals in Table 1. It is interesting to note that nickel (Ni) and cobalt (Co) show particularly high negative S-values with −19 and −20 µV/K, respectively. No S-values are given for the metal alloys, as they vary for different compositions.

**Figure 1:**
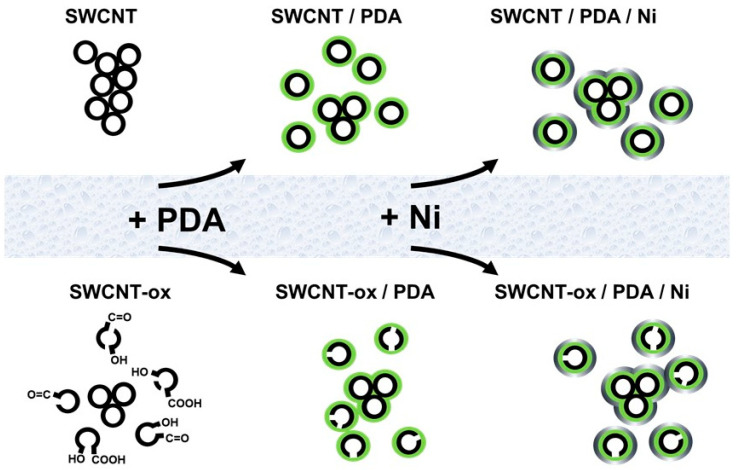
Schematic drawing of the SWCNTs; process steps to functionalize and metallize yielding in structure modification.

The aim of the present study is to develop a technique for coating SWCNTs with nickel using PDA as an adhesive coupler to investigate how the coating affects their thermoelectric properties (Figure 1). If possible, the Seebeck coefficients of the SWCNTs should be reversed to generate n-type SWCNTs. To the best of our current information, PDA-functionalized and electroless nickel-coated SWCNTs have not been fabricated yet as hybrid nanoparticles. Both the modification of the SWCNTs with PDA and the subsequent nickel deposition take place in an aqueous medium. Unmodified SWCNTs are difficult to disperse in water due to their polarity. Therefore, oxidized SWCNTs were investigated as a parallel approach in the modification steps. They are better dispersible and separable in water due to their significantly more polar character. The research study will investigate whether the two surface modifications lead to higher electroless metal deposition rates when the SWCNTs are more strongly separated at the beginning of the synthesis. The influence of all modification steps on their thermoelectric properties will be investigated.

## 2. Materials and Methods

### 2.1. Materials

Single-walled carbon nanotubes (SWCNTs) of the type Tuball™ grade 75% (OCSiAl Europe S.à r.l., Leudelingen, Luxembourg) with diameters less than 2 nm and lengths larger than 1 μm were used as electrically conductive filler. Structural details are described in [56]. Tuball™ SWCNTs were selected because earlier investigations showed that this material has a very high Seebeck coefficient and is suitable for changing the thermoelectric conduction type [57,58].

Nitric acid (AnalaR NORMAPUR Nitric Acid, 65%, VWR International GmbH, Darmstadt, Germany) was used to oxidize the SWCNTs, and dilution deionized water (ELGA PURELAB Plus, Veolia Water Technologies Deutschland GmbH, Celle, Germany) was applied.

### 2.2. SWCNT Treatment

#### 2.2.1. SWCNT Oxidation

Three grams of SWCNTs was added to 150 mL of HNO_3_ (AnalaR NORMAPUR Nitric Acid, 65%, VWR International GmbH, Darmstadt, Germany) and dispersed in an ultrasonic bath USC600TH (VWR International GmbH, Darmstadt, Germany) at 120 W for 10 min. The reaction mixture was boiled for 2 h under reflux, then cooled with ice water and diluted with 100 mL deionized water via the reflux condenser. The SWCNTs were separated by suction filtration using a PTFE filter with a 1.2 µm pore size (Sartorius Stedim Biotech, Göttingen, Germany) and washed neutrally. Before further use, the SWCNTs were dried for 8 h at 120 °C in a vacuum oven. The oxidized SWCNTs appear in the paper as “SWCNT-ox”.

#### 2.2.2. Dispersion and PDA Deposition

For TRIS buffer preparation, 10 mmol/L 2-amino-2-(hydroxymethyl)propane-1,3-diol (99.9%, Roche Diagnostics GmbH, Grenzach-Wyhlen, Germany) was dissolved in deionized water (0.055 µS/cm) and adjusted to pH = 8.5 using hydrochloric acid (37%, VWR International GmbH, Darmstadt, Germany).

Briefly, 0.3 g of SWCNT or SWCNT-ox was dispersed in 750 mL of TRIS buffer. An ultrasonic processor (UP400St, Hielscher Ultrasonics GmbH, Teltow, Germany) with a sonotrode H3 (Hielscher Ultrasonics GmbH, Teltow, Germany) was used for vigorous mixing at an amplitude of 60% with a cycle of 1 for 5 min.

The SWCNT and SWCNT-ox dispersions were diluted with an additional 1.25 L of TRIS buffer. Then, 4 g of dopamine hydrochloride solution (2 g/L, 99%, Thermo Fisher GmbH, Bremen, Germany) (DA) was added. The suspensions were stirred at 250 rpm for 60 min to allow DA oxidative polymerization to endow the nanotube surfaces with PDA. Afterwards, they were suction filtered using PTFE filters with a 1.2 µm pore size and washed twice with 100 mL of deionized water.

#### 2.2.3. Metallization of the PDA-SWCNTs

An electroless nickel plating bath series from MacDermit Enthone, Waterbury, CT, USA, was used to metallize the CNTs. The SWCNT/PDA and SWCNT-ox/PDA sample dispersions were first immersed in a colloidal palladium activator bath (UDIQUE 879W) for 3 min at 30 °C under stirring, then filtered off by suction filtration using PTFE filters and washed two times with deionized water (2× 100 mL). Next, CNTs were immersed in an accelerator bath solution (UDIQUE 8810) for 2.5 min at 50 °C with stirring, then separated from the solution by suction filtration with PTFE filters. In the metal coating bath process, the SWCNT/PDA and SWCNT-ox/PDA were metallized using a nickel-plating bath (UDIQUE 891, UDIQUE 892, UDIQUE 893) for 8 min at 30 °C. The pH of the nickel bath was adjusted to pH 9 with ammonium hydroxide (28–30 wt% solution of ammonia in water: NH_3_⋅H_2_O, Acros Organics B.V.B.A., Geel, Belgium). After metallization, the CNTs, now named SWCNT/PDA/Ni and SWCNT-ox/PDA/Ni, were separated from the solution by suction filtration with PTFE filters, washed twice with deionized water (2× 100 mL), and dried under vacuum at 25 °C for 30 min.

### 2.3. Characterization

For the transmission electron microscopy (TEM) study, a drop of the freshly prepared aqueous dispersion was placed on a carbon-coated TEM grid and dried in air. The TEM images were collected with a Libra120 (Carl Zeiss GmbH, Oberkochen, Germany).

For scanning electron microscopy (SEM), the dry powder was put on the grid. A Carl Zeiss Ultra plus SEM with an SE2 detector at 3 kV (Carl Zeiss Microscopy Deutschland GmbH, Oberkochen, Germany) was used for these studies. Before imaging, the surfaces were coated with 3 nm platinum.

All XPS studies were carried out by means of an Axis Ultra photoelectron spectrometer (Kratos Analytical, Manchester, UK). The spectrometer was equipped with a monochromatic Al Kα (h⋅ν = 1486.6 eV) X-ray source of 300 W at 15 kV. The kinetic energy of the photoelectrons was determined with a hemispheric analyzer set to pass energy of 160 eV for wide-scan spectra and 20 eV for high-resolution spectra. With Scotch double-sided adhesive tape (3M Company, Maplewood, MN, USA), the powdery SWCNT samples were prepared as thick films on a sample holder, enabling the samples’ transport to the recipient of the XPS spectrometer. During all measurements, electrostatic charging of the sample was avoided by means of a low-energy electron source working in combination with a magnetic immersion lens. Later, all recorded peaks were shifted by the same value that was necessary to set the component peak *Gr* showing the sp^2^-hybridized carbon atoms of the graphite-like lattice (–*^Gr^*C=*^Gr^*C– ↔ =*^Gr^*C–*^Gr^*C=) to 283.99 eV [59]. Quantitative elemental compositions were determined from peak areas using experimentally determined sensitivity factors and the spectrometer transmission function. Spectrum background was subtracted, according to Shirley [60]. The high-resolution spectra were deconvoluted by means of the Kratos spectra deconvolution software (Vision Processing, version 2.2.9 [2011], provided by Kratos Analytical, Manchester, UK). Free parameters of component peaks were their binding energy (BE), height, full width at half maximum, and the Gaussian–Lorentzian ratio.

For the AFM investigation, freshly cleaved HOPG (highly oriented pyrolytic graphite) was dipped into CNT-water suspensions for 5 s. The remaining drops were removed by a paper tissue. The AFM measurements were done in tapping mode using a Dimension FastScan AFM (Bruker-Nano, Billerica, MA, USA) and silicon nitride sensors with a sharpened silicon tip (ScanAsyst-Fluid+) (Bruker-Nano, Billerica, MA, USA). They have a nominal spring constant of 0.7 N/m and a nominal resonance frequency of 150 kHz, and the tip radius is 2 nm. Height images (surface morphology) and phase images were taken simultaneously. According to Magonov [61], the scan conditions were chosen either to get stiffness contrast (free amplitude > 100 nm, setpoint amplitude ratio 0.8) or adhesion contrast (free amplitude < 20 nm, setpoint amplitude ratio 0.7) in the phase image.

The thermoelectric (TE) characterization was carried out using a Seebeck measuring device developed at IPF Dresden. More details are given in [57,62]. The measurements were performed at 40 °C with temperature differences between the two copper electrodes up to 8 K. For the measurements on powders, the SWCNTs were filled into a double T-shaped sample consisting of a PVDF tube (inner diameter 3.8 mm, length 16 mm) sealed with copper plugs (see Figure 1 in [57]). The measurement of voltage and resistance was performed using the Keithley multimeter DMM2001 (Keithley Instruments, Cleveland, OH, USA) as a 4-wire technique for powders. The values given represent the mean values of three measurements.

## 3. Results

### 3.1. Dispersion Stability

**Figure 2:**
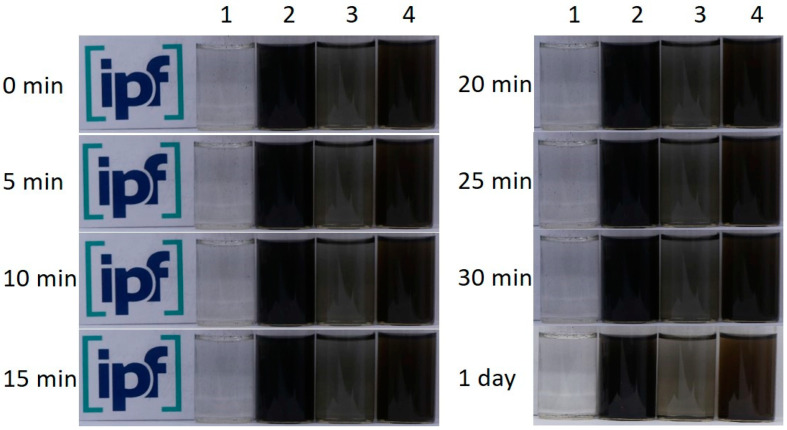
Sedimentation stability over one day. Photographs of aqueous dispersion of SWCNT (1), SWCNT-ox (2), SWCNT/PDA (3), SWCNT-ox/PDA (4).

The stability of the aqueous SWCNT dispersions was observed over a longer period of time (Figure 2). It can be clearly seen that the oxidation of the SWCNTs leads to a significantly more homogeneous and stable SWCNT dispersion. For the as-grown SWCNTs, agglomerates can be seen at the bottom of the glass while the solution remains clear. The fine dispersion of SWCNT-ox appears dark grey and remains like this for 24 h. The dispersions containing PDA already appear grey-brown due to the PDA (samples 3 and 4), but the dispersion with SWCNT-ox is darker than the dispersion with as-grown SWCNT and also stable over a longer time. It is known from the literature that the oxidation of CNTs leads to the formation of polar groups on the surface, followed by a significant increase in their polarity, which thus improves their dispersibility in polar water [63,64,65]. From the stability test, it can be concluded that the PDA coating of oxidized SWCNTs leads to the best SWCNT distribution in polar medium water, which is advantageous for the next process step of nickel deposition.

The high dispersion stability of both SWCNT types coated with PDA is a prerequisite for good accessibility of the SWCNT surface in the metallization process. While functionalization with an organic material does not change their properties much, metallization creates a dense nickel shell (Ni density is about 8.9 g/cm³). In comparison, the density of plain SWCNTs is assumed to be around 1.5 g/cm³ for SWCNTs with an outer diameter of 2 nm [66]. Consequently, the properties of the nanomaterials, i.e., surface/volume ratio and density, as well as dispersion and sedimentation behaviour are significantly affected by the nickel shell (compare Figure 1). Nickel-coated SWCNTs settle to the bottom as sediment.

### 3.2. Morphological Characterisation

**Figure 3:**
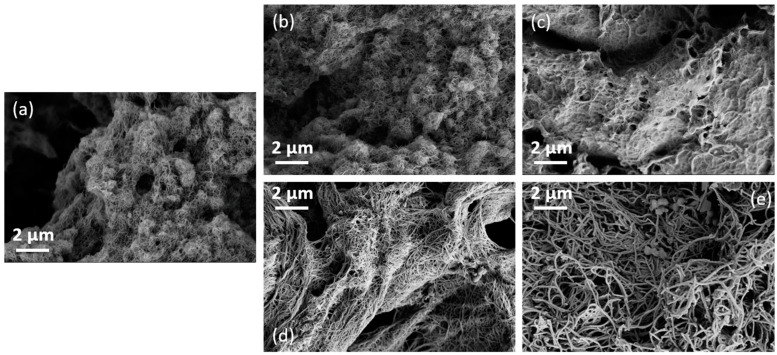
SEM image of SWCNT unmodified (**a**) SWCNT/PDA (**b**), SWCNT/PDA/Ni (**c**), and SWCNT-ox /PDA (**d**), SWCNT-ox/PDA/Ni (**e**).

In Figure 3, the SEM images of the differently modified SWCNTs are shown. In the case of the as-grown SWCNTs, the PDA coating (Figure 3b) hardly leads to any change in the appearance of the SWCNTs (Figure 3a). In the SWCNT-ox/PDA sample (Figure 3d), the SWCNTs appear thicker and more separated than in the SWCNT/PDA sample (Figure 3b). After Ni coating, the SWCNT/PDA/Ni appears like a glued mat of SWCNTs (Figure 3c). In contrast, the SWCNT-ox/PDA/Ni (Figure 3e) can be recognized as individual fibres that are significantly thicker than the SWCNT-ox/PDA. Also, some spherical particles are visible. In summary, the coating of the oxidized SWCNT with PDA and nickel resulted in a more homogeneous material.

**Figure 4:**
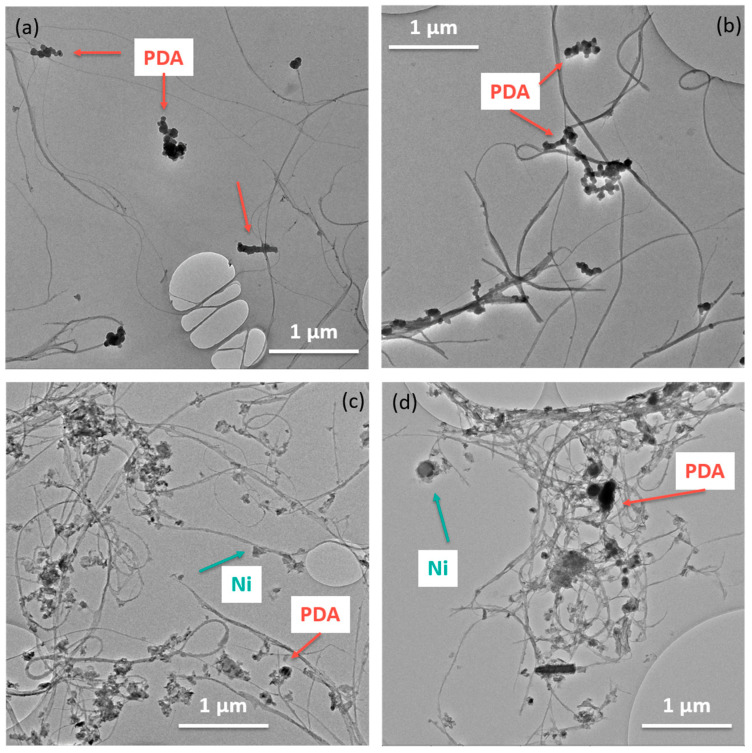
TEM image of SWCNT/PDA (**a**) and SWCNT-ox /PDA (**b**), SWCNT/PDA/Ni (**c**), SWCNT-ox/PDA/Ni (**d**).

In Figure 4a,b, the TEM images of both SWCNT types coated with PDA are shown. It is assumed that the black spherical particles in the images are PDA agglomerates from the aqueous dispersion. It seems that the PDA particles preferentially attach to the oxidized SWCNTs, while in the case of the non-functionalized SWCNTs, the PDA particles tend to lie next to the SWCNTs. The thin PDA coating cannot be reliably detected for any SWCNT type, even at higher magnifications. The TEM images of both SWCNT types coated with PDA and Ni (Figure 4c,d) show small grey areas adhering to the SWCNTs. It is assumed that this is nickel. For both SWCNT types, nickel always seems to be associated with the SWCNTs. Whether the SWCNTs were evenly coated cannot be estimated from the images.

**Figure 5:**
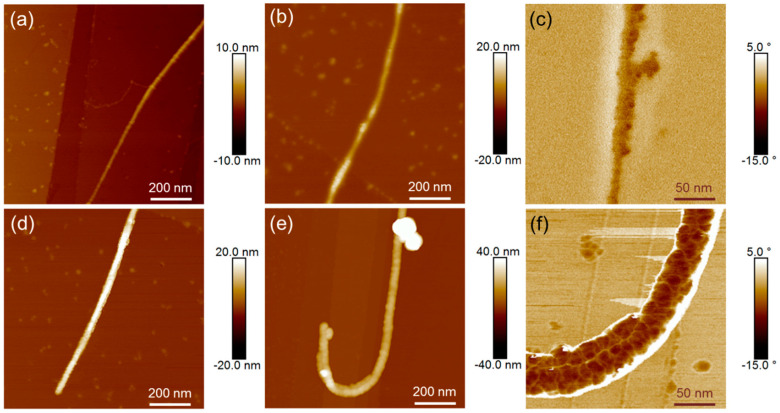
Topography images of individual SWCNTs: SWCNT (**a**), SWCNT-ox (**d**), SWCNT/PDA (**b**), SWCNT-ox/PDA (**e**); phase images of PDA-modified SWCNTs at higher magnification: SWCNT /PDA (**c**), SWCNT-ox/PDA (**f**).

Figure 5 shows AFM images of individual pristine, oxidized, and PDA-coated SWCNT. A comparison of pristine and oxidized CNTs is difficult because the effect of the oxidation is on the atomic scale and therefore below the resolution of the AFM. The PDA layer is hardly visible on the non-oxidized SWCNTs, neither in the topography (Figure 5b) nor in the phase image (Figure 5c). On a contrary, topography and phase images of the PDA-coated oxidized SWCNTs (Figure 5e,f) clearly show the characteristic globular structure of the PDA layer.

**Figure 6:**
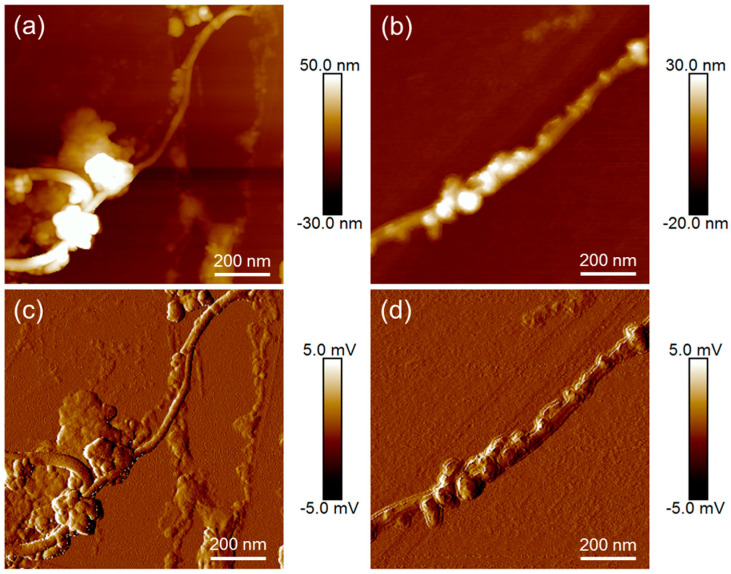
Topography images (**a**,**b**) and amplitude error images (**c**,**d**) of metallized SWCNTs: SWCNT/PDA/Ni (**a**,**d**), SWCNT-ox/PDA/Ni (**b**,**d**).

In Figure 6, AFM images of PDA- and Ni-coated SWCNTs are compared. Besides the height images, amplitude error images are presented. They show the first deviation of the height and, therefore, small structures. It is clearly visible that on oxidized fibres with a more homogeneous PDA coating (Figure 6b,d) the Ni distribution is more uniform, while on unoxidized PDA-coated SWCNTs (Figure 6a,c) the Ni is arranged in clusters.

### 3.3. Investigation of the Chemical Composition and Bonding States near the Surface

**Figure 7:**
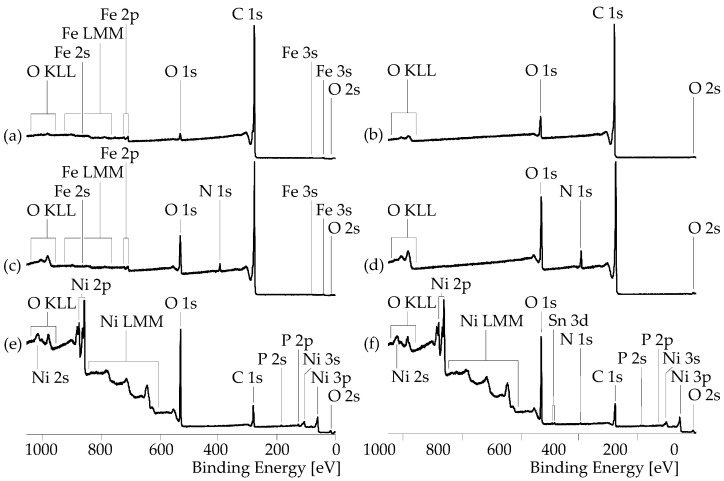
XPS wide-scan spectra recorded from pristine SWCNT (**a**), SWCNT-ox (**b**), SWCNT/PDA (**c**), SWCNT-ox/PDA (**d**), SWCNT/PDA/Ni (**e**), and SWCNT-ox/PDA/Ni (**f**) samples.

**Figure 8:**
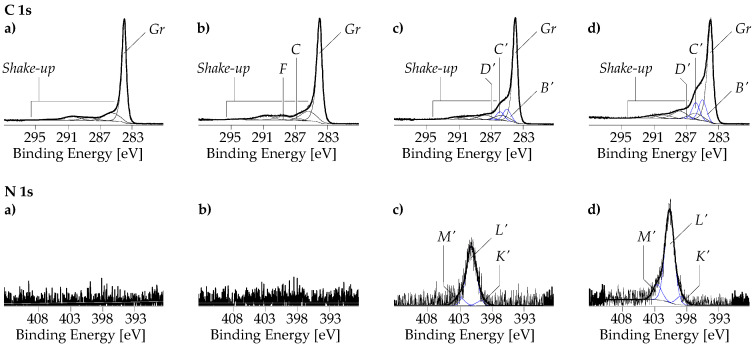
C 1s and N 1s high-resolution XPS spectra recorded from pristine SWCNT (**a**), SWCNT-ox (**b**), SWCNT/PDA (**c**), and SWCNT-ox/PDA samples. The blue component peaks in the figures (**c**) and (**d**) demonstrate the presence of PDA on the SWCNT surfaces (their origins).

As can be seen in the XPS wide-scan spectrum, the pristine SWCNT sample contains only traces of oxygen ([O]:[C] = 0.019) (Figure 7a). The majority of this oxygen is probably bound to iron, which was also clearly detected in the sample as Fe 2s, Fe 2p, and Fe LMM Auger series. No component peaks for oxidized carbon species could be separated in the high-resolution C 1s spectrum (Figure 8a). The main component peak *Gr* in the C 1s spectrum at 283.99 eV results from photoelectrons of the sp^2^-hybridized carbon atoms of the graphite-like lattices in their electronic ground states. Photoelectrons from electronically exited states produced by π → π* transitions were collected as shake-up peaks on the spectrum’s high-energy side.

The oxidation of the SWCNTs increased not only the relative oxygen content significantly to [O]:[C] = 0.056, but it also removed the iron impurities in the sample (Figure 7b). The component peaks *C* (at 286.54 eV) and *F* (at 288.33 eV) separated in the C 1s spectrum (Figure 8b) showed that oxidation of the carbon took place and that mainly phenolic C–OH groups (component peak *C*) and carboxylic acid groups (component peak *F*) were introduced in the surfaces of the SWCNT-ox sample.

Deposition of PDA creates N 1s peaks and enhances the oxygen peaks in the spectra. Taking its intensity as a measure of the PDA content in the surface region of the SWCNTs, it can be seen that the oxidation had a beneficial effect on the adsorption and interfacial polymerization of the DA (Figure 7c,d). It increased the relative nitrogen content from [N]:[C] = 0.036 for the SWCNT/PDA sample to [N]:[C] = 0.06 for the SWCNT-ox/PDA sample, i.e., about twice the amount of PDA was deposited on the hydrophilic SWCNT-ox surface. The corresponding C 1s and N 1s spectra (Figure 8c,d) show the characteristic component peaks indicating the presence of PDA on the SWCNT surfaces. In addition to the main component peaks *Gr* and the shake-up peaks, we found the component peaks *B*′ (at ca. 285.11 eV), *C*′ (at ca. 285.97 eV), and *D*′ (at ca. 287.25 eV). Component peaks *B*′, which have twice the intensity as the [N]:[C] ratios, show the C–N bonds of the PDA’s indoline and indole units. Component peaks *C*′ result from the catechol groups (*^C^*^′^C–OH). Since some of the catechol groups were present in their oxidized form, namely as quinone-like groups, their intensities were slightly smaller than the intensities of peaks *B*′. The quinone-like groups (*^D^*^′^C=O) were represented by the component peaks *D*′. The main component peaks *L*′ (at ca. 399.7 eV) in the N 1s spectra show the fractions of the PDA’s indoline-like bonded nitrogen atoms (C–*^L^*^′^NH–C). Due to the increased electron density of the indole-bonded nitrogen atoms (C=*^K^*^′^N–C ↔ C–*^K^*^′^N=C), the component peaks *K*′ were shifted to lower binding energy values (ca. 398.03 eV). Protonated secondary amino groups (C–*^M^*^′^N^⊕^H_2_–C) were identified as component peaks *M*′ at ca. 401.58 eV.

After the metallization, the element peaks of nickel (Ni 2s, Ni 2p, and Ni LMM *Auger* series) appear in the wide-scan spectra (Figure 7e,f). Surprisingly, the relative nickel content of SWCNT/PDA with lower PDA content was with [Ni]:[C] = 0.509, significantly greater than that of the SWCNT-ox/PDA sample with more PDA in the SWCNT/nickel interphase ([Ni]:[C] = 0.305). This is surprising at first glance but can be explained by the different distribution of the nickel visible in the SEM images (Figure 3). On the SWCNT/PDA/Ni sample, a lot of metal is deposited between the CNTs, while on the SWCNT-ox/PDA/Ni sample, only the CNTs are covered with nickel.

**Figure 9:**
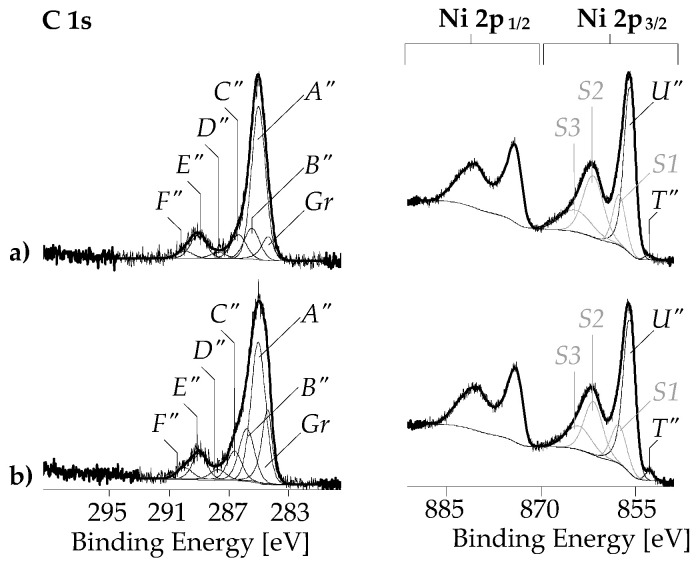
C 1s and Ni 2p high-resolution XPS spectra recorded from SWCNT/PDA/Ni (**a**), and SWCNT-ox/PDA/Ni (**b**) samples. (The origins of the component peaks are explained in the text).

The almost complete disappearance of the N 1s peaks together with the changed shapes of the C 1s spectra (Figure 9a,b) shows that the PDA film is mostly covered by the nickel layer. The presence of carbon is now largely due to the presence of surface contaminants, such as fatty acid esters that spontaneously adsorb onto metal oxides and minimize their surface-free energy. Detailed analysis shows, however, that the nickel layers are not fully closed. In the deconvolution process of the spectra, it was necessary to introduce the component peak *Gr* (at ca. 284.3 eV), which indicates the presence of graphite-like bonded carbon atoms in the surface region. The intense component peaks *A*″ (at 285.00 eV) result from photoelectrons from sp^3^-hybridized carbon atoms that have no heteroatoms in their immediate vicinity. Carboxylic ester groups are identified by the component peaks *C*″ (alcohol-sided carbon atoms, O=C–O–*^C^*C) and *E*″ (carbonyl carbon atoms, O=*^E^*C–O–C). Component peaks *F*″ arise from carboxylic acid groups (O=*^F^*^″^C–OH) and their corresponding carboxylates (O=*^F^*^″^C–O^⊖^ ↔ ^⊖^O–*^F^*^″^C=O). Carbon atoms in α-position to the carbonyl carbon atoms (*^B^*^″^C–COO) are observed as component peaks *B*″. The small component peaks *D*″ at 287.7 eV are an indication of the presence of ketone groups (O=*^D^*^″^C).

The complex shapes of the Ni 2p spectra (Figure 9c,d) and the high relative oxygen contents (Figure 7e,f) indicate that nickel is present preferentially in its oxidized forms (formally as Ni^2+^). The positions of the main component peaks *U*″ in the high-resolution Ni 2p_3/2_ spectra at ca. 855.64 eV confirm the presence of NiO and/or Ni(OH)_2_ [67]. The small component peaks *T*″ on the low-energy side of the main component peak result from metallic nickel (Ni^0^). The divalent nature of the nickel ions (Ni^2+^) mainly present in the samples corresponds to the [Ar]4s^0^3d^8^ electron configuration with a magnetic moment of 2.83 Bohr magneton. The photoionization of this electron configuration results in the [1s^2^2s^2^2p^6−1^3s^2^3p^6^]4s^0^3d^8^ electron state, which can be neutralized by electrons from the oxygen-containing ligands (L) situated in the nickel’s coordination sphere. The so-called *local screening* resulted in the [1s^2^2s^2^2p^6−1^3s^2^3p^6^]4s^0^3d^9^[L^−1^] electron ground state causing the component peaks *U*″ mentioned above. An electronically excited state resulted from a second electron transfer from the ligands to Ni^2+^. Photoelectrons from this [1s^2^2s^2^2p^6−1^3s^2^3p^6^] 4s^0^3d^10^[L^−2^] state are observed as satellite peaks (*S2*) at ca. 861.35 eV. Oxygen-containing ligands that are further away can also provide electrons for the photoionized nickel ions via a transfer by hopping (non-local screening to the [1s^2^2s^2^2p^6−1^3s^2^3p^6^]4s^0^3d^9^[L][3d^8^L^−1^] state). The corresponding satellite peaks (*S1*) are found at ca. 857.46 eV. Finally, satellite peaks *S3* arising at ca. 863.57 result from photoelectrons in the [Ar]4s^0^3d^8^ state, which are not neutralized by the electrons provided by the ligands.

### 3.4. Thermoelectric Characterisation

**Figure 10:**
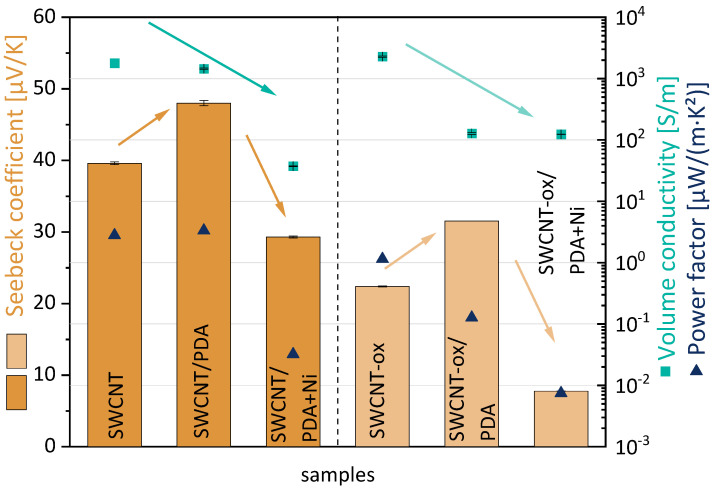
Thermoelectric parameter of SWCNTs at different modification state (the green and brown arrows only serve to illustrate the trends).

In Figure 10 the values of volume conductivity, Seebeck coefficient, and power factor are summarized for the three different modification states of SWCNT and oxidized SWCNT. The Seebeck coefficient of pristine SWCNT powder is determined to be 39.6 µV/K [57]. The oxidation of the SWCNT leads to a lower S-value of 22.4 ± 0.1 µV/K. It is assumed that the insertion of hydroxy and carboxyl groups partially destroys the perfect structure of the SWCNT walls and thus impairs electron transport across the SWCNT walls, resulting in a lower S-value. At the same time, SWCNT oxidation leads to an increase in volume conductivity from 1790 S/m [57] up to 3291 ± 59 S/m. Thus, the PF decreases from 2.8 [57] to 1.1 µW/m·K² due to the lower Seebeck coefficient.

For both as-grown SWCNTs and oxidized SWCNTs, the PDA coating leads to an increase in the S-value by 9 µV/K, such that S-values of 48.0 ± 0.4 µV/K (SWCNT) and 31.5 ± 0.0 µV/K (SWCNT-ox) are achieved, i.e., PDA acts as a p-dopant agent. It is surprising that the effect is about the same for SWCNTs and SWCNT-ox because both morphological examination and XPS showed a thicker and more homogeneous PDA coating on the SWCNT-ox. In addition, after PDA coating, the electrical conductivity of SWCNT-ox powders drops sharply to 128 ± 4 S/m, and only slightly for SWCNTs (1451 ± 32 S/m). The electrically insulating PDA layer can presumably deteriorate electron transport by direct contact with the SWCNTs or even electron hopping. Thus, the PDA modification leads to a slightly higher PF for SWCNT/PDA at 3.3 µW/m·K² compared to SWCNT. However, for the SWCNT-ox, the PF decreases from 1.1 µW/m·K² (SWCNT-ox) to 0.1 µW/m·K² (SWCNT-ox/PDA) due to the strongly decreased volume conductivity.

The coating of both SWCNT/PDA types with nickel leads to a reduction of the Seebeck coefficient to 29.3 ± 0.1 µV/K (SWCNT) and 7.8 ± 0.0 µV/K (SWCNT-ox). This reduction is consistent with the negative S-value of the nickel itself of −19 µV/K [48,62]. Nickel thus acts as an n-dopant, as expected. This effect is clearly more pronounced with the oxidized SWCNTs. Here, the S-value is reduced by 23.8 µV/K instead of only 18.7 µV/K for SWCNT/PDA. Obviously the more uniform nickel coating of the oxidized SWCNTs, as seen in Figure 3, has a stronger effect on the S-value despite the lower amount of deposited nickel revealed by XPS. Unfortunately, the amount of nickel deposited was not sufficient to produce SWCNTs with a negative Seebeck coefficient. The XPS study showed that the nickel layer is not closed on the SWCNT surface. Thus, p- and n-conducting structures alternate. Presumably, this is one reason why no negative S-value was obtained. Thus, the PF decreases for both samples to 0.03 µW/m·K² (SWCNT) and 0.01 µW/m·K² (SWCNT-ox).

## 4. Discussion

The present study investigates how SWCNTs can be coated with nickel using PDA as an adhesive coupler in a “green” process in aqueous solutions. With regard to thermoelectric applications, the aim was to reverse the Seebeck coefficient of the SWCNTs to negative values since nickel as a metal has a negative S-value of −19 µV/K.

Oxidation of the SWCNTs improved their dispersibility in water. The better dispersibility of the oxidized SWCNTs remained after modification of the SWCNTs with PDA, which led to better modification results.

Both pristine and oxidized SWCNTs were modified with dopamine and metallized with nickel. XPS verified the deposition of PDA and nickel on both pristine and oxidized SWCNTs. While more PDA was detected on oxidized SWCNTs, the elemental ratio of nickel was significantly higher on non-oxidized SWCNTs. This corresponds to morphology studies of the SWCNTs carried out by SEM, TEM, and AFM. On the surfaces of oxidized SWCNTs, PDA and nickel are homogeneously distributed. On non-oxidized SWCNTs, however, no continuous PDA layer was detected, and nickel was deposited not only on the CNTs but also in the spaces between them.

The thermoelectric characterisation shows that PDA acts as a p-dopant, resulting in an increase in the Seebeck coefficient by around 9 µV/K for both pristine and oxidized SWCNT. This increase was not helpful with regard to the aim of the study (reduction of the S-value), but the PDA coating was only an intermediate step. The coating with nickel successfully reduced the S-value by 19 µV/K (SWCNT/PDA) and 23.8 µV/K (oxidized SWCNT/PDA). The deposited amount was, however, not sufficient to create a negative Seebeck coefficient.

The results show that electroless metallization is able to create a nickel layer on PDA-modified SWCNTs. Prior oxidation of the SWCNTs is essential to ensuring good dispersion and homogeneous coating with PDA and nickel. The nickel coating can actually change the conduction type of the SWCNTs, but more work is needed to adjust the nickel layer thickness.

In future studies, the thermoelectric properties will be investigated as a function of Ni content or Ni layer thickness on the SWCNT. On the other hand, it is interesting that the PDA coating increases the S-value. Here, too, the dependence between the PDA coverage of the SWCNTs and their thermoelectric properties is worth studying.

## Figures and Tables

**Table 1:** Selected electrolessly depositable metals, their Seebeck coefficients, and metal alloys for main industrial applications.

Metals	Reference	Seebeck Coefficient S [µV/K] @300 K	Metal Alloys	Reference
Ni	[5,10,11,23,43,44,45,46,47]	−19 [48]	CuNi, NiCo, PdNiP, NiWP,	[11,43]
Cu	[11,43,49,50,51]	1.7 [48]	CuNi, CuCo, CuAu, CuCd,	[11]
Co	[11]	−1.7 [52]	CuCo, NiCo, CuCd, CuAu, PdCoP	[11]
Cd	[11]	2.6 [13]	CuCd,	[11]
Ag, Ag-NP *	[9,11,43,53]	1.5 [13]	AgAu,	[11]
Au	[11,43]	1.9 [13]	AuSn, AgAu, CuAu, AuIn	[11,43]
Pt	[11]	−4.9 [48]		
Pd	[11]	−10.7 [13]	PdCoP, PdZnP, PdNiP,	[11]
Rh	[11]	1 [54]		
Cr	[11]	12 [54]		
Zn	[11]	2.4 [13]	ZnCo, NiZn, PdZnP	[11]
Sn	[11,43]	−1 [55]	AuSn, SnPb	[11]
Co	[11,43]	−20 [54]	CuCo, PdCoP,	[11]

* nanoparticle (NP).

## Data Availability

The data presented in this study are available upon request from the corresponding author.
